# Agonist and antagonist effects of tobacco-related nitrosamines on human α4β2 nicotinic acetylcholine receptors

**DOI:** 10.3389/fphar.2015.00201

**Published:** 2015-09-22

**Authors:** Simone Brusco, Paola Ambrosi, Simone Meneghini, Andrea Becchetti

**Affiliations:** Department of Biotechnology and Biosciences, University of Milano-BicoccaMilano, Italy

**Keywords:** CHRNA4, CHRNB2, HEK, nAChR, NNK, NNN, partial agonist, patch-clamp

## Abstract

Regulation of the “neuronal” nicotinic acetylcholine receptors (nAChRs) is implicated in both tobacco addiction and smoking-dependent tumor promotion. Some of these effects are caused by the tobacco-derived N-nitrosamines, which are carcinogenic compounds that avidly bind to nAChRs. However, the functional effects of these drugs on specific nAChR subtypes are largely unknown. By using patch-clamp methods, we tested 4-(methylnitrosamine)-1-(3-pyridyl)-1-butanone (NNK) and N'-nitrosonornicotine (NNN) on human α4β2 nAChRs. These latter are widely distributed in the mammalian brain and are also frequently expressed outside the nervous system. NNK behaved as a partial agonist, with an apparent EC_50_ of 16.7 μM. At 100 μM, it activated 16% of the maximal current activated by nicotine. When NNK was co-applied with nicotine, it potentiated the currents elicited by nicotine concentrations ≤ 100 nM. At higher concentrations of nicotine, NNK always inhibited the α4β2 nAChR. In contrast, NNN was a pure inhibitor of this nAChR subtype, with IC_50_ of approximately 1 nM in the presence of 10 μM nicotine. The effects of both NNK and NNN were mainly competitive and largely independent of V_m_. The different actions of NNN and NNK must be taken into account when interpreting their biological effects *in vitro* and *in vivo*.

## Introduction

The nAChRs are ligand-gated ion channels permeable to cations, which regulate cell excitability and synaptic transmission. They are formed by different α and β subunits, which assemble to form homo- or heteropentamers (Dani and Bertrand, 2007). The homopentameric receptors, such as the widespread (α7)_5_, have low sensitivity to ACh and nicotine (with EC_50_ higher than 100 μM), high permeability to Ca^2+^ (P_Ca_) and rapid desensitization. The heteromeric nAChRs generally display higher sensitivity to the agonists, lower P_Ca_ and slower desensitization (Dani and Bertrand, 2007). The most common high-affinity form in the mammalian brain is the α4β2^*^, with apparent EC_50_ values at least 10 times lower than those displayed by (α7)_5_. In the peripheral nervous system, the prevalent nAChR subtype is α3β4, which shows a more restricted expression in the CNS (Zoli et al., 2015). Nicotinic subunits are also widely expressed in other tissues, where their functions are still debated (Wessler and Kirkpatrick, 2008). In cancer cells, different types of homo- and heteromeric nAChRs cooperate in controlling several aspects of the neoplastic phenotype (Egleton et al., 2008; Schuller, 2009; Ambrosi and Becchetti, 2013; Schaal and Chellappan, 2014). Recently, the nAChR genes CHRNA3, CHRNA5, and CHRNB4 have been implicated in lung cancer susceptibility as well as nicotine addiction (Improgo et al., 2013, and references therein).

**Figure 1:**
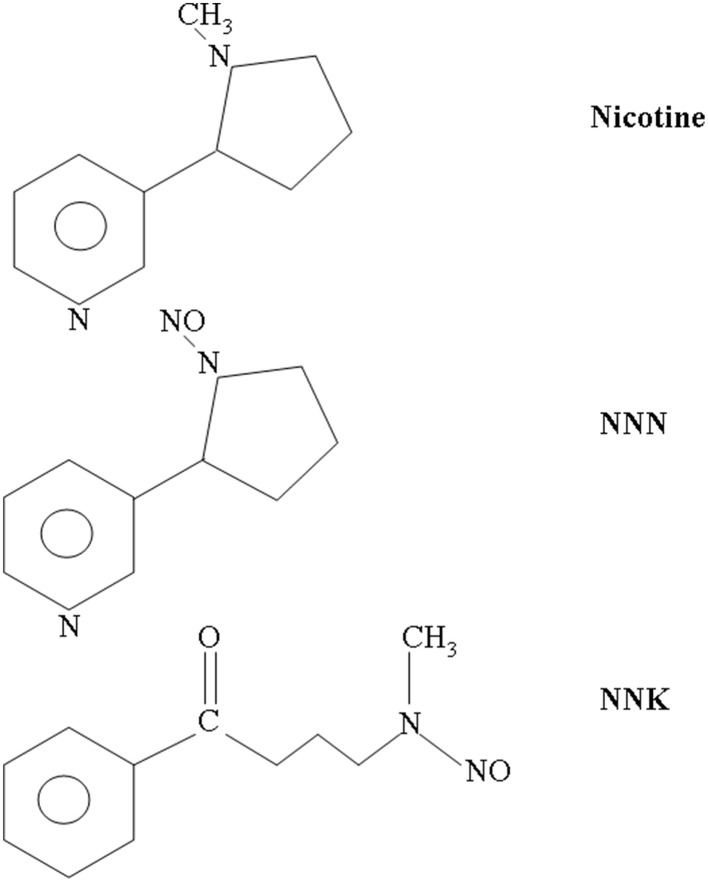
**Molecular structures of nicotine, NNK, and NNN**. NNN and NNK are the two most potent carcinogenic N-nitrosamines. They derive from nitrosation of nicotine, which occurs during tobacco treatment and storage as well as through metabolic processing in mammals.

The complex pattern of nAChR expression may partly explain the pleiotropic effects of smoking. In the brain, uncontrolled stimulation of nAChRs is thought to mediate the addictive effects of tobacco (Changeux, 2010). In other tissues, smoking can produce toxic as well as carcinogenic effects. These latter depend on long-term exposure to several tobacco metabolites, especially the N-nitrosamines (here simply referred to as “nitrosamines”; Hecht and Hoffmann, 1988). When metabolically activated, these drugs cause DNA mutations, particularly G to T transversions that may lead to mutation of k-ras and p53 (Pfeifer et al., 2002). Nonetheless, many harmful effects of the tobacco-related nitrosamines may be attributed to direct targeting of nAChRs. In fact, the nitrosamines that produce the most potent biological effects are structural analogs of either ACh (e.g., diethylnitrosamine) or nicotine (especially NNK and NNN; Figure 1). All of these compounds can bind to nAChRs (Schuller and Orloff, 1998; Schuller, 2007). Although nicotine is not carcinogenic, engagement of nAChRs with either nicotine or nitrosamines can promote the neoplastic progression in cultured cells, by stimulating cell cycle, migration, angiogenesis, epithelial-to-mesenchymal transition, and inhibiting apoptosis (Schuller, 1989; Maneckjee and Minna, 1990; Heeschen et al., 2001; Arredondo et al., 2006; Guo et al., 2008; Paleari et al., 2008; Song et al., 2008; Al-Wadei and Schuller, 2009; Calleja-Macias et al., 2009; Dasgupta et al., 2009). Similar evidence is slowly accumulating *in vivo* (Heeschen et al., 2001; Paleari et al., 2009).

The pathophysiological interpretation of these studies requires to clarify the signaling pathways downstream to nAChR binding (West et al., 2003; Tsurutani et al., 2005; Guo et al., 2008; Ambrosi and Becchetti, 2013). However, a general hindrance to full comprehension of the cellular effects of nitrosamines is that the direct functional action of these drugs on nAChRs is largely unknown. The only such study currently available shows that NNN inhibits α3β4 nAChR at high concentrations (IC_50_ was 1.14 mM, in the presence of 100 μM nicotine), whereas it exerts little effect on the muscle subtype (Nunes-Alves et al., 2012). The lack of direct functional tests is a major lacuna, as binding studies alone cannot distinguish whether a certain compound directly activates or inhibits nAChRs, nor the related kinetics. Moreover, the downstream cell signals could be regulated by conductive as well as non-conductive mechanisms (Becchetti, 2011). Understanding the functional interplay between nitrosamines and ACh or nicotine is also necessary to interpret their effects in the brain. Smokers are exposed to a mixture of tobacco-related nAChR ligands. Compounds such as NNK easily traverse the blood brain barrier (Jorquera et al., 1992; Gerde et al., 1998; Berridge et al., 2010) and have been found to activate microglia *in vivo* (Ghosh et al., 2009). These molecules are likely to interfere with one another and with the physiological agonist, to alter synaptic transmission and regulate tobacco addiction in currently unpredictable ways.

We studied by patch-clamp methods the effect of NNK and NNN on human α4β2 nAChRs stably expressed in HEK cells. This nAChR subtype is widely distributed in the brain, where it regulates both excitatory and inhibitory transmission (Becchetti et al., 2015) and is implicated in the cognitive and addictive effects of smoking (Changeux, 2010; Faure et al., 2014). It is also commonly expressed in non-nervous tissue, including lung cells (Fu et al., 2009), and tumor cell lines (Egleton et al., 2008). We found that these nitrosamines exert distinct actions, as NNK is a partial agonist, whereas NNN is a pure inhibitor of α4β2 nAChRs.

## Materials and methods

### Cell cultures

HEK 293 cells stably expressing human α4β2 nAChRs were cultured by standard methods (Di Resta et al., 2010). In brief, cultures were maintained in Dulbecco's modified Eagle's medium (Hyclone Laboratories), supplemented with 10% fetal calf serum (Hyclone), 4.5 g/l glutamine, 0.05 ng/ml hygromycin β, and 0.25 μg/ml amphoterycin B. Cells were grown at 37°C and 5% CO_2_. For patch-clamp experiments, cells were harvested by treatment with trypsin and plated onto 35 mm Petri dishes (Corning Inc.).

### Patch-clamp recording

Cells were voltage-clamped by using the whole-cell configuration of the patch-clamp method. Currents were registered with an Axopatch 200B amplifier (Molecular Devices), at room temperature (20–22°C). Micropipettes (3–4 MΩ) were pulled from borosilicate capillaries (GMBH) with a P-97 Flaming/Brown Micropipette Puller (Sutter Instrument Co.). The cell capacitance and series resistance (up to about 75%) were always compensated. Currents were low-pass filtered at 2 kHz and acquired on-line at 5–10 kHz with Molecular Devices hardware and software (pClamp 8 and Axoscope 8). After patch rupture, we usually allowed 1–2 min for pipette solution exchange and signal stabilization, before applying our stimuli. Extracellular solutions were applied with an RSC-160 Rapid Solution Changer (BioLogic Science Instruments). The solution is delivered to the cells through borosilicate capillaries connected to Tygon tubes. Seven independent lines are available. Each line was generally reserved to one compound at a given concentration. The perfusing line was totally substituted whenever the drug was changed. No corrections for leak or junction potentials was ever applied to any of the displayed results.

### Solutions and drugs

Unless otherwise indicated, chemicals, and drugs were purchased by Sigma-Aldrich Italia Srl. The extracellular solution contained (mM): NaCl 130, KCl 5, CaCl_2_ 2, MgCl_2_ 2, HEPES 10, D-glucose 5 (pH 7.4; adjusted with NaOH). Pipettes contained (mM): K-aspartate 120, NaCl 10, MgCl_2_ 2, CaCl_2_1.3, EGTA-KOH 10, HEPES 10, MgATP 1, (pH 7.3; adjusted with KOH). Stock solutions of nicotine (up to 10 mM) were prepared weekly in our external solution and kept refrigerated. The pH was always checked after nicotine addition. NNK and NNN (20 mM) were dissolved in water and kept refrigerated for no longer than 1 week. Nicotine and/or nitrosamines were added daily to the extracellular solution. The final concentrations were obtained by serial dilution.

### Analysis of data

Data were analyzed with Clampfit 9.2 (Molecular Devices) and OriginPro 9 (OriginLab Co.). Theoretical curves best fitting the data were determined by a nonlinear least-squares method (Levenberg-Marquardt algorithm). For nAChR activation we used both a simple Hill function and the sum of two Hill expressions (Covernton and Connolly, 2000). The single-term expression was:

(1) $$\begin{array}{l} \frac{I_{L}}{I_{\max}}=\frac{1}{{\left(1+\frac{EC_{50}}{\left[L\right]}\right)}^{n_{H}}} \end{array}$$

where *I*_max_is the maximal current, *I*_L_ is the peak current at a given concentration of agonist *L*, EC_50_ is the concentration of *L* at which *I*_*L*_/*I*_max_ = 0.5, and *n*_H_ is the Hill coefficient.

The two-terms Hill expression was:

(2) $$\begin{array}{l} \frac{I_{L}}{I_{\max}}=\frac{A}{{\left(1+\frac{EC_{50high}}{\left[L\right]}\right)}^{n_{H1}}}+\frac{1-A}{{\left(1+\frac{EC_{50low}}{\left[L\right]}\right)}^{n_{H2}}} \end{array}$$

where *A* is the fraction of receptors in the high-affinity state; EC_50high_ and EC_50low_ are the EC_50_ values of the components at high and low affinity, respectively; *n*_H1_ and *n*_H2_ are the corresponding Hill coefficients, and the other symbols are as in Equation (1).

The function used to fit the desensitization data points (**Figure 3**) was:

(3) $$\begin{array}{l} \frac{I_{SS}}{I_{peak}}=B+\frac{1-B}{{\left(1+\frac{\left[L\right]}{IC_{50}}\right)}^{n_{H}}} \end{array}$$

where *I*_SS_ is the average steady state current at a given concentration of agonist *L, I*_peak_is the corresponding peak current,*B* is the minimal fractional steady state current, IC_50_ is the concentration of *L* at which *I*_SS_/*I*_peak_ = 0.5, and *n*_H_ is the Hill coefficient. *I*_SS_ was found by fitting the current decay with a single exponential function and taking the steady state value of this function. A similar function was used to fit the fractional inhibition in **Figures 4B, 5B**.

The function used to fit the concentration-response data for nicotine at a given NNk concentration (**Figure 5C**; red continuous line) was:

(4) $$\begin{array}{l} y=\frac{\alpha_{Nic}\frac{\left[Nic\right]}{EC_{50}}+\frac{\alpha_{NNK}\left[NNK\right]}{EC_{50\left(NNK\right)}}}{1+\frac{\left[Nic\right]}{EC_{50}}+\frac{\left[NNK\right]}{EC_{50\left(NNK\right)}}} \end{array}$$

where *y* is the fractional current in the presence of NNK plus nicotine, α_Nic_and α_NNK_are the maximal fractional responses produced by nicotine and NNK, respectively (also named intrinsic activities of the agonists; Hogg and Bertrand, 2007); [Nic] and [NNK] are, respectively, the concentrations of nicotine and NNK; EC_50_ and EC_50(NNK)_ are, respectively, the EC_50_ values for nicotine and NNK. The model is simplified in that it only considers single EC_50_'s for both the full and the partial agonist.

### Statistics

Data are given as mean values ± standard error of the mean, with *n* indicating the number of determinations (i.e., the number of cells tested). Statistical significance was determined with two-tailed *t*-test for paired or unpaired samples, as appropriate, at the indicated significance level (*p*). Normality was tested by the Kolmogorov-Smirnov test.

## Results

### NNK activates human **α**4**β**2 nAChRs

**Figure 2:**
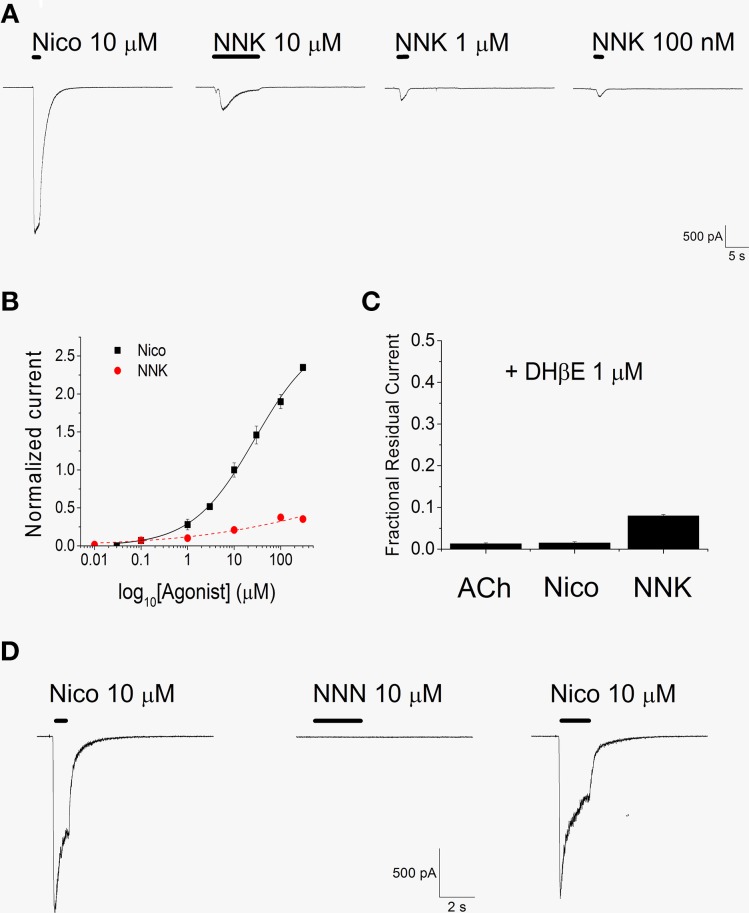
**NNK, but not NNN, activates α4β2 nAChRs**. **(A)** Consecutive whole-cell current traces, elicited at -80 mV by the indicated compound. Tests were spaced 2–3 min apart (gaps in the continuous current trace). Continuous bars above the current traces indicate the time of agonist application. Nicotine was repeatedly applied during the experiment to check for possible current rundown. **(B)** Concentration-response relations for nicotine (black squares) and NNK (red circles). Data points are average peak whole-cell currents recorded at −80 mV, normalized to the current elicited by 10 μM nicotine and plotted against the agonist concentration (in Log_10_ scale). Each point is the average of at least seven determinations. The concentration-response relation for nicotine (continuous line) and that for NNK (dashed line) were fitted with Equation 1, which gave an EC_50_ = 27.8 ± 5.6 μM (*n*_*H*_ = 0.64) for nicotine and 16.7 ± 20 μM (*n*_*H*_ = 0.44) for NNK. The estimated maximal current activated by NNK was approximately 16% of the current elicited by 300 μM nicotine. The measured peak current in the presence of 100 μM NNK was 0.14 ± 0.02 (*n* = 13) of the value measured in the presence of 300 μM nicotine. **(C)** DHβE (1 μM) strongly blocked the currents elicited by 300 μM ACh, 300 μM nicotine, and 10 μM NNK, as indicated. Data are averages of at least seven determination for each condition, carried out at −80 mV. Bars display the fractional residual currents (i.e., the peak current measured in the presence of the agonist plus DHβE divided by the peak current activated by the agonist alone). In the presence of 10 μM NNK, DHβE brought the average peak current density (pA/pF) from 5.75 ± 1.22 to 0.53 ± 0.199 (*p* < 0.01 with paired *t*-test; *n* = 9). **(D)** Comparison of the effects of NNN (10 μM) and nicotine (10 μM) on whole-cell currents from α4β2 nAChRs, at −80 mV. NNN never produced nAChR activation in cells in which functional receptors were shown to be present by nicotine application.

We first tested if NNK can activate α4β2 nAChRs, by applying the drug at −80 mV. A representative example is shown in Figure 2A. Consecutive applications of different NNK concentrations were spaced 2–3 min apart, to permit full recovery from nAChR desensitization. NNK concentrations higher than 10 nM elicited inward currents, and the maximal effect was obtained at 100 μM NNK. Nicotine was usually applied at the beginning and at the end of the experiment, to check for possible current rundown. The concentration-response relation for NNK was obtained by plotting the average fractional peak currents obtained at the indicated NNK concentrations (Figure 2B). On average, the current elicited by 100 μM NNK was approximately 14% of the current activated by saturating concentrations of nicotine (300 μM). No significantly higher currents were observed when using 300 μM NNK. For both nicotine and NNK, data points were fitted with Equation 1, which gave apparent EC_50_'s of approximately 28 μM for nicotine and 17 μM for NNK. For all kinetic parameters, detailed statistics are given in the figure legends, which also report the Hill coefficients. For easier consulting, the main results are summarized in Table 1.

**Table 1:** **Comparison of the effects of nicotine, NNN, and NNK, on α4β2 nAChRs**.

**Compound**	**I_max_/I_nico_**	**EC_50_ (μM)**	**EC_50*high*_**	***EC*_50*low*_**	**Desensitization IC_50_ (μM)**	**Inhibition IC_50_ (μM)**
			**(μM)**			
Nicotine	1	27.8 (*n*_*H*_ = 0.65)	0.14 (*n*_*H*1_ = 1.1)	7.7 (*n*_*H*2_ = 2.1)	2.64 (*n*_*H*_ = 1.8)	N.A.
NNK	0.16	16.7 (*n*_*H*_ = 0.44)	0.035(*n*_*H*1_ = 1.2)	12.81 (*n*_*H*2_ = 1.4)	1.7 (*n*_*H*_ = 0.9)	>100 (with 10 μM nicotine)
NNN	0	N.A.	N.A.		N.A.	0.00021 (with 10 μM nicotine)

The current activated by NNK was strongly blocked by 1 μM dihydro-β-erythroidine (DHβE), which is known to inhibit α4β2, but not homomeric, nAChRs (Buisson et al., 1996; Alkondon et al., 2000). To allow equilibration of DHβE concentration, this compound was perfused in the bath for 30 s before the agonist was added. To generate the data shown in Figure 2C, the peak current measured in the presence of agonist plus inhibitor was divided by the one elicited by the agonist alone. These fractional residual currents were plotted for NNK (10 μM), nicotine (300 μM), and ACh (300 μM). DHβE blocked more than 90% of the currents activated by either agonist, which is consistent with our hypothesis that NNK specifically activated the α4β2 nAChRs expressed in our cells.

**Figure 3:**
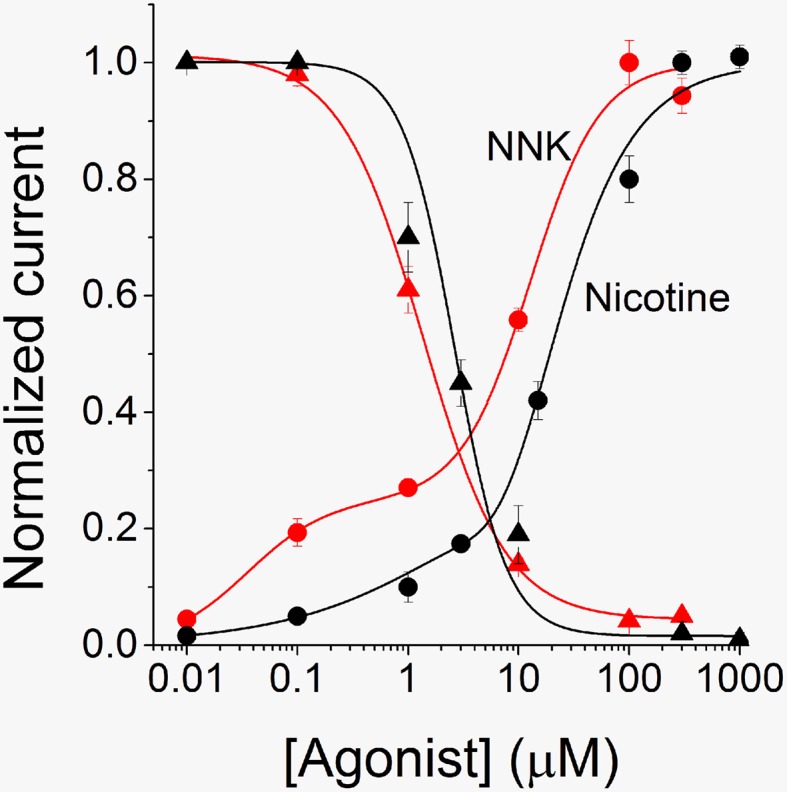
**Activation and desensitization profiles for α4β2 nAChRs**. The activation curve for NNK (red circles) was generated by using experiments analogous to those shown in Figure 2. The average peak current measured at each concentration of NNK was normalized to the current elicited by 300 μM NNK. Data points are averages of at least seven determinations and were fitted by using Equation 2 (continuous line), which gave EC_50high_ = 0.035 ± 0.012 μM (*n*_H2_ = 1.2), and EC_50low_ = 12.81 ± 1.58 μM (*n*_H1_ = 1.4). The desensitization curve for NNK was generated by plotting average steady state fractional currents (red triangles), as a function of NNK concentration. At each concentration, the current decay in the presence of the drug was fitted with a single exponential function. The steady state current values thus estimated were divided by the corresponding peak current values. Data points are averages of at least 6 determinations and were fitted by using Equation 3 (continuous line), which gave IC_50_ = 1.7 μM ± 0.2 (*n*_*H*_ = 0.9). The nicotine activation (black circles) and desensitization (black triangles) curves were obtained in a similar way. For activation, data points are averages of at least 6 determinations. They were fitted by using Equation 2 (continuous line), giving EC_50high_ = 0.14 ± 1.03 μM (*n*_H2_ = 0.62), and EC_50low_ = 7.7 ± 14.7 μM (*n*_H1_ = 1.8). For desensitization, data points were fitted with Equation 3 (continuous line), which gave IC_50_ = 2.64 ± 0.47 μM (*n*_H_ = 1.84).

A more detailed analysis of the concentration-response relations for nicotine and NNK is shown in Figure 3. Best fitting of the experimental data points was obtained by using the two-terms Hill function (Equation 2). This is usually observed with α4β2 nAChRs (Covernton and Connolly, 2000; Buisson and Bertrand, 2001), and is attributed to the coexistence of two receptor's stoichiometries: (α4)_3_(β2)_2_ (with lower affinity) and (α4)_2_(β2)_3_ receptors (with higher affinity; Nelson et al., 2003). The fitting parameters were: EC_50high_ = 0.035 μM and EC_50low_ = 12.81 μM, for NNK, and EC_50high_ = 0.14 μM and EC_50low_ = 7.7 μM for nicotine. These results suggest that NNK is particularly effective at stimulating the high affinity nAChR component. Moreover, for both nicotine (e.g., **Figure 5A**) and NNK (e.g., Figure 2A), the activated current displayed progressive desensitization in the presence of the agonist. To quantify such process, the current decay was fitted with a monoexponential function (e.g., Paradiso and Steinbach, 2003). Next, desensitization curves were generated by plotting the average fractional steady state currents calculated from the fitting procedure, for the indicated agonist concentration (Figure 3). By using Equation 3 (continuous lines through the data points), we estimated IC_50_ = 1.7 μM for NNK, and IC_50_ = 2.64 μM for nicotine. In the absence of other nicotinic ligands, the nAChR-dependent biological effect of NNK must depend on the steady state current sustained by this drug. This is proportional to the product of the activation and desensitization curves (sometimes referred to as “window current”), at a given NNK concentration. Figure 3 suggests that the window currents of NNK and nicotine are broadly similar, but that NNK tends to be comparatively more effective at the low concentrations, in line with the range of plasma doses observed in smokers.

### The inhibitory effect of NNN

**Figure 4:**
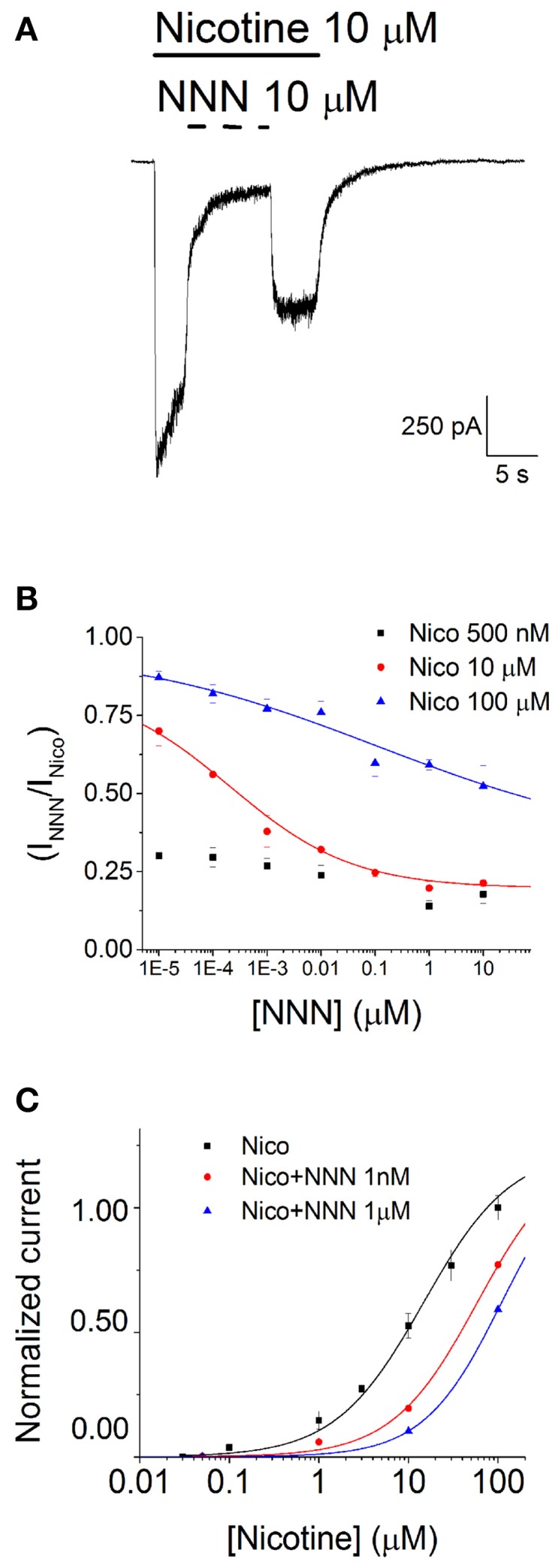
**NNN inhibits α4β2 nAChRs**. **(A)** Typical whole-cell current traces elicited by 10 μM nicotine at −80 mV, in the presence and in the absence of 10 μM NNN. Horizontal bars mark time of application of the indicated compound. **(B)** Steady state inhibition curves were generated by plotting the residual fractional steady state currents as a function of NNN concentration (in Log_10_ scale). The different data sets were obtained at the indicated concentration of nicotine. Data points are averages of at least nine determinations. Lines through the data points are best fitting curves, obtained with Equation 3. At 100 μM nicotine, IC_50_ was > 20 μM, whereas at 10 μM nicotine IC_50_ was 0.21 ± 0.4 nM. **(C)** Activation curves in the absence (black squares) or in the presence of the indicated concentration of NNN. Data points are peak whole-cell currents normalized to the current elicited by, 100 μM nicotine. Continuous lines through the data points are best fitting curves, obtained with Equation 1. The corresponding parameters were: EC_50_ = 14.5 ± 1.34 μM (*n*_H_ = 0.83), for nicotine alone; EC_50_ = 58.3 ± 4.4 μM (*n*_H_ = 0.92), in the presence of 1 nM NNN; EC_50_ = 109.1 ± 0.14 μM (*n*_H_ = 1), in the presence of 1 μM NNN.

Differently from NNK, NNN did not elicit any whole-cell current even at concentrations (100 μM) much higher than those encountered in physiological conditions. Figure 2D shows a typical experiment at−80 mV, comparing the effects of 10 μM nicotine and 10 μM NNN, in a cell expressing a large nicotinic current. Once again, nicotine was applied before and after NNN, to exclude channel rundown. Given that NNN produced no nAChR activation, we studied its possible inhibitory effect in the presence of nicotine. We tested NNN concentrations between 1 pM and 100 μM on currents activated by concentrations of nicotine ranging between 10 nM and 100 μM. A representative current trace is shown in Figure 4A, in which 10 μM NNN was applied in the presence of 10 μM nicotine, at −80 mV. NNN was generally applied until the effect had reached the steady state. Next, the drug was removed. After the current had recovered from inhibition, nicotine was also washed out. This experimental procedure (Buisson et al., 1996; Palma et al., 1996) was preferred to the alternative procedure of pre-conditioning with NNN and then applying simultaneously the agonist and the antagonist (as we did with DHβE). The former method allows to directly compare the effect of NNN with the one produced by nicotine. In this way, the possible artifacts occurring in repetitive consecutive trials (such as channel rundown, poor solution exchange, precise estimation of the current peak, etc.) are avoided or immediately recognized. Moreover, the blockade kinetics can be directly appreciated. For each concentration, the steady state current in the presence of NNN was divided by the current remaining after NNN was removed. These fractional currents were plotted in Figure 4B, as a function of the NNN concentration, for the indicated nicotine concentrations (0.5, 10, and 100 μM). Notice that higher nicotine concentrations decreased the inhibitory effect of NNN, which suggests a competitive blocking mechanism. For instance, the IC_50_ value for NNN was >10 μM in the presence of 100 μM nicotine, whereas it was approximately 1 nM in the presence of 10 μM nicotine (full statistics are reported in the figure legend). Conversely, the concentration-response relations for nicotine in the presence of the indicated NNN concentrations are plotted in Figure 4C. Data points were fitted with Equation 1 (continuous lines through the data points). The right-shift of the curves produced by NNN is consistent with the notion that this drug tends to produce competitive block of α4β2 nAChRs. From a pathological point of view, it is worth noticing that NNN can exert significant current block at concentrations normally encountered in smokers' plasma (<100 pM; Schuller, 2007).

### The effect of NNK in the presence of nicotine

**Figure 5:**
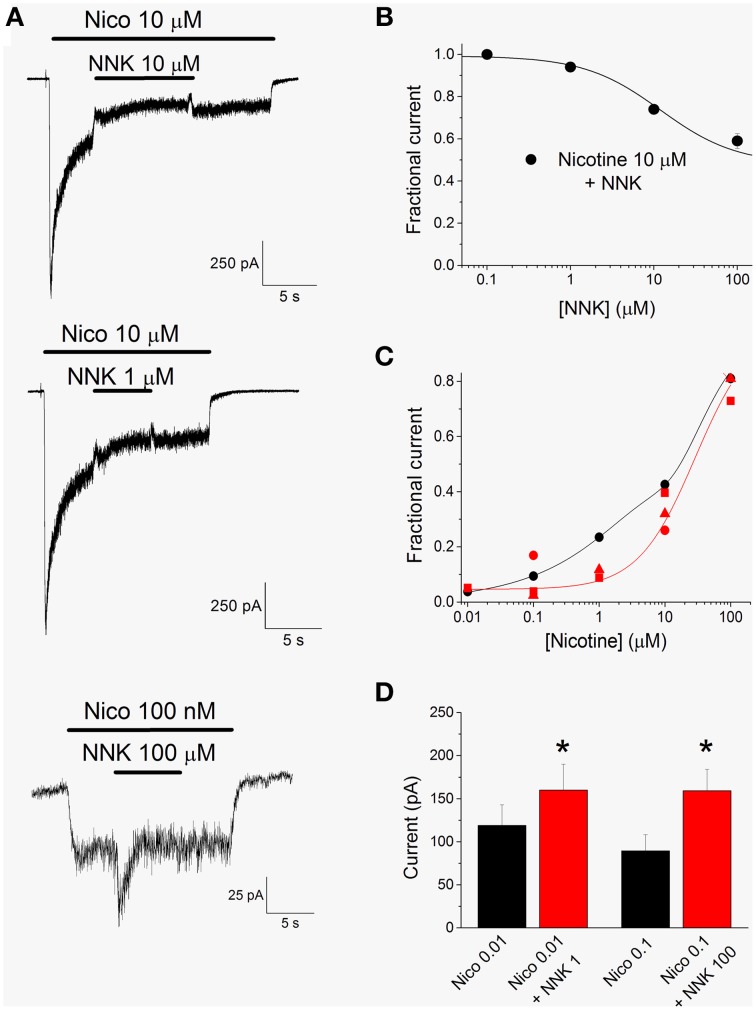
**The effect of NNK on α4β2 nAChRs, in the presence of nicotine**. **(A)** Representative current traces elicited at −80 mV by the indicated concentration of nicotine (Nico), in the presence or in the absence of the indicated concentration of NNK. The currents elicited by 10 μM nicotine (top and middle panels) are representative of 11 similar experiments. The bottom panel illustrates the potentiating effect of 100 μM NNK on the current activated by 100 nM nicotine (representative of eight similar experiments). Bars mark the time of application of nicotine and NNK, as indicated. **(B)** Concentration-response relation for the inhibitory effect of NNK tested on currents activated by 10 μM nicotine, as illustrated in **(A)**. Data points are average steady state currents (*n* = 11) measured in the presence of a given NNK concentration, divided by the current obtained after NNK was rinsed. Continuous line through the data points is best fitting to Equation 3, giving IC_50_ > 100 μM (*n*_H_ = 0.95). **(C)** Summary of the effects of NNK plus nicotine. All data are normalized to the current measured with 300 μM nicotine. Black circles: concentration-response for nicotine (same as in Figure 3; only concentrations up to 100 μM are shown). Red symbols: average fractional currents in the presence of 1 μM (squares), 10 μM (triangles), or 100 μM (squares) NNK, as a function of [nicotine]. Data were obtained as illustrated in **(A)**. The values obtained with NNK were generally significantly different from those obtained with nicotine alone. Detailed statistics for the potentiation data are given in **(D)**. The red continuous line is the curve best fitting the data points relative to 1 μM NNK, obtained by using Equation 4. The fit parameters were *A* = 1, *B* = 0.27, [NNK] = 1 μM, α_NIC_ = 24 μM, α_NNK_ = 5 μM. **(D)** The peak current measured in the presence of 10 nM nicotine was 119 ± 24 pA, which was brought to 160 ± 30 pA by 1 μM NNK (0.05 < *p* < 0.01, with *t*-test for paired samples; *n* = 11). The effect of 100 μM NNK was tested in a different series of cells, in which the average current elicited by 100 nM nicotine was 89.4 ± 19.4 pA, which was brought to 159.1 ± 25.1 pA by 100 μM NNK (0.05 < *p* < 0.01, with *t*-test for paired samples; *n* = 8). These results are plotted as black and red bars, respectively for nicotine (Nico) and NNK. Statistical significance is indicated by ^*^.

NNK is expected to produce effects more complex than those shown by NNN, as partial agonists can produce channel activation or inhibition depending on the concentration of the full agonist (e.g., Hogg and Bertrand, 2007; Rollema et al., 2007). In fact, concentrations of NNK up to 100 nM produced no effect on the currents activated by 10 μM nicotine, whereas higher concentrations progressively inhibited α4β2 nAChRs (Figure 5A). These data were obtained and analyzed as previously illustrated for NNN. Figure 5B plots the average fractional residual currents measured in the presence of 10 μM nicotine, as a function of NNK concentration. Data points were fitted with Equation 3 (continuous line), giving an IC_50_ > 100 μM. In contrast, the currents activated by low concentrations of nicotine could be potentiated by NNK. An example is given in the bottom panel of Figure 5A, showing that 100 μM NNK increased the current activated by 100 nM nicotine at −80 mV, by approximately 80%. In agreement with the results shown in Figure 3, the current activated by 100 μM NNK also displayed progressive desensitization. The effects of the tested concentrations of NNK on the currents activated by different concentrations of nicotine are summarized in Figure 5C. For comparison, the activation curve for nicotine (black circles) is also reported. With 1 μM NNK (red squares), the potentiation produced on the currents activated by 0.01 μM nicotine was 26%. The concentration of nicotine at which the drug reversed its effect was around 20 nM, as is estimated by fitting the data points with the simplified model expressed by Equation 4 (continuous red line). Saturating concentrations of NNK (100 μM; red circles) potentiated by about 80% the current activated by 0.1 μM nicotine, whereas they inhibited by 40% the current elicited by 10 μM nicotine. In this case, the sign reversal of NNK effect can be estimated to occur at approximately 400 nM nicotine. For clarity, the error bars are not reported in Figure 5C. Instead, the statistics of the current potentiation observed with NNK are reported in detail in Figure 5D and in the figure legend. We conclude that NNK, consistently with its partial agonist nature, can produce either potentiation or inhibition of α4β2 nAChRs, depending on the concomitant concentration of the full agonist.

### The block produced by NNN and NNK was not voltage-dependent

**Figure 6:**
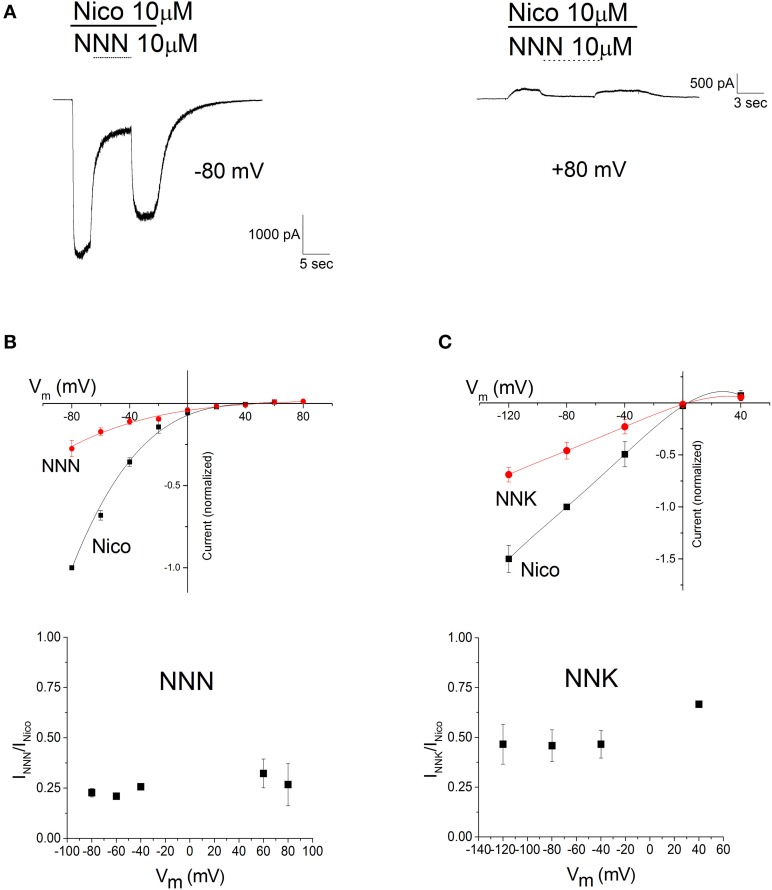
**The effects of NNN and NNK are not voltage-dependent**. **(A)** Typical current traces obtained as illustrated in Figure 3, except that trials were carried out at different V_m_'s. For briefness, only the traces obtained at −80 and +80 mV are displayed. Bars mark time of application of 10 μM nicotine (Nico, continuous) and 10 μM NNN (dashed). **(B)**. Top panel: current-voltage relations obtained in the absence (squares) and in the presence (circles) of NNN. Data points are average steady state current densities measured at the indicated V_m_ and normalized to the value obtained at -80 mV (with a reversed sign, for consistency with the usual convention of displaying inward current as negative). Data summarize the results of nine independent experiments. Continuous lines are polynomial curves best fitting the data points. No correction was applied for junction potentials. Bottom panel: the fractional block produced by NNN is plotted as a function of V_m_. Data points are average steady state currents in the presence of NNN (I_NNN_), divided by the current in the absence of NNN (I_Nico_). The data points around V_rev_ were omitted (see the main text). No significant difference was observed among the results obtained at different V_m_'s. **(C)** Top panel: same as in **(C)**, for NNK (1 μM, in the presence of 100 nM nicotine). Bottom panel: the fractional block produced by NNK as a function of V_m_ was calculated as illustrated for NNN in **(B)**, except that the tested V_m_'s were: −120/−80/−40/0/+40. Once again, no significant difference was observed among the results obtained at different V_m_'s.

The voltage dependence of the NNN and NNK effect is illustrated in Figure 6. Current traces (Figure 6A) illustrate typical experiments in which nAChRs were activated by 10 μM nicotine, at the indicated V_m_. For briefness, only traces obtained at −80 mV (left) and +80 mV (right) are shown. NNN (10 μM) was applied in the presence of nicotine and removed after inhibition had reached the steady state. These results are summarized in Figure 6B (top panel), showing the current voltage relations in the presence of either nicotine alone (Nico) or nicotine plus NNN (NNN). Data points are average current values normalized to the absolute value of the current measured at −80 mV. The I/V plots displayed the typical inward rectification of α4β2 nAChRs. Analogous results were obtained with NNK (Figure 6C, top panel). The apparent reversal potential (V_rev_) was usually between +5 and +20 mV. The fractional block produced by either NNN or NNK at the steady state was independent of the applied V_m_. This is better appreciated in the bottom panels of Figures 6B,C, which give the average fractional residual currents as a function of V_m_, in the presence of NNN or NNK, respectively. The data points around V_rev_ were removed as the small current values prevented a reliable measure of the drugs' effect. The quick development and reversal of channel block suggests that the effect was mainly caused by the nitrosamines accessing to the channel from the extracellular *milieu* (i.e., we assume that intracellular accumulation of the drugs was negligible). Hence, because inhibition was virtually independent of the net direction of ion flow, these nitrosamines are unlikely to exert significant open channel block, at the concentrations we applied (much higher than those observed *in vivo*).

## Discussion

### Comparison with previous studies

Binding of nitrosamines to nAChRs was previously studied with radioactive ligand competition assays. These were mostly carried out in lung cancer cell lines, by using labeled epibatidine (to target heteromeric nAChRs) or α-bungarotoxin (specific for α7; Schuller and Orloff, 1998; Schuller, 2007). Our results qualitatively agree with these studies in that our nitrosamines compete with the full agonist for binding to nAChRs. However, a detailed quantitative comparison is difficult to draw. First, tumor cell lines generally express multiple nAChR subtypes (Egleton et al., 2008), and these have different pharmacological properties (e.g., Dani and Bertrand, 2007). Second, electrophysiological and binding analyses address different molecular features that can be difficult to reconcile (e.g., Chang and Weiss, 1999). For example, patch-clamp measurements reveal the population of active channels, whereas the binding profile can be affected by silent receptors, whose level of expression may be significant (McNerney et al., 2000). Even with those qualifications, the EC_50_ we observed for NNK (Figure 3) with the single-term Hill model is in good agreement with the one measured in binding studies carried out on epibatidine-sensitive nAChRs (about 17 μM, in SCLC cells; Schuller, 2007). The highest binding efficacy of NNN on α4β2 nAChRs is also in broad agreement with the binding studies. The fact that NNN inhibited α4β2 nAChRs (Figure 4) is consistent with the functional studies carried out on α3β4 receptors, although in the latter the affinity is lower and the mechanism is mainly non-competitive (Nunes-Alves et al., 2012). Based on our results and the absence of open channel block (Figure 6), we hypothesize that the main site of nitrosamine action on α4β2 nAChRs is close to the orthosteric binding site.

### Functional implications for peripheral and cancer tissue

Work on cell lines showed that nicotine and nitrosamines produce similar effects, particularly stimulation of proliferation and inhibition of apoptosis. In SCLC cells and gastrointestinal cancer, the effects are mainly attributed to the activation of α7 receptors, whereas in other neoplastic cells, such as the NSCLC, both homo- and heteromeric nAChRs contribute to the observed effects (Schuller, 2009). In brief, nAChR activation tends to stimulate exocytosis of autocrine factors (Cattaneo et al., 1993; Jull et al., 2001) as well as other Ca^2+^-dependent pathways that regulate proliferation and apoptosis (Schuller, 2009). The relative contribution of the different nAChR subtypes is still debated. One general working hypothesis is that, differently from α7 receptors, activation of heteromeric nAChRs stimulates pathways that tend to suppress cancer progression (Schuller, 2009). If this hypothesis is correct, our results suggest that NNN could stimulate tumor progression by inhibiting the protective effects contributed by heteromeric nAChRs, without affecting the less sensitive α7. Alternatively, NNN might exert signaling roles not depending on ion conduction (e.g., Dasgupta et al., 2006), or activate other types of membrane receptors, or both. Evidence is indeed available about the activation produced by NNK on β-adrenergic receptors (Schuller et al., 1999), but not about NNN, to the best of our knowledge.

The partial agonist nature of NNK makes it more difficult to suggest a general interpretation of its cellular effects, particularly because the direct effects of NNK on α7 nAChRs are currently unknown. Considering that the binding studies indicate that homomeric receptors are more sensitive to NNK, a reasonable working hypothesis is that NNK induces both homo- and heteromeric nAChR activation in cultured cells, and that the overall effect on V_m_ and calcium signals tends to prevail on the tumor suppressing signals specifically induced by α4β2 activation. Moreover, although neither α7 (Kawai and Berg, 2001) nor α4β2 (Buisson and Bertrand, 2001) receptors display long-term inactivation in the presence of nicotine, the long-term effects of nitrosamines on nicotinic currents are unknown.

Even more caution is necessary to interpret the effects of the tobacco-related nitrosamines *in vivo*. We showed that the typical concentrations of these compounds observed *in vivo* can produce functional effects on α4β2 nAChRs. Smokers' blood contain steady nitrosamine levels around 30–50 pM, and the peak concentrations can be at least 10 times as high (e.g., Hecht and Hoffmann, 1988; Schuller, 2007). In parallel, smokers are exposed to widely fluctuating levels of plasma nicotine, depending on smoking habits, not to speak of the concomitant physiologically oscillating ACh levels depending on parasympathetic activity. How nitrosamines and the full agonists interfere in single individuals is difficult to predict, given that the typical peak concentration of plasma nicotine in smokers generally vary between 10 and 500 nM (e.g., Russell et al., 1980), but can reach concentrations as high as 10 μM, immediately after smoking (Schaal and Chellappan, 2014). According to our results, this wide range of concentrations allows both potentiating and inhibiting effects of NNK, at least for α4β2 nAChRs. These observations may contribute to explain the individual variability in the response to long-term smoking. In general, our study points to the necessity of carrying out more extensive work *in vitro* on the combined effects of nitrosamines and the full agonists on specific nAChR subtypes.

### Implication for brain pathophysiology

Because of their slow desensitization and high sensitivity to ACh, α4β2 nAChRs are major regulators of the overall cerebral excitability, as is also testified by the observation that the nAChR-related epileptogenic mutations known to date are located on genes coding for subunits of heteromeric receptors (Becchetti et al., 2015). In the mammalian neocortex, heteromeric (largely α4β2^*^) nAChRs regulate excitatory (Vidal and Changeux, 1993; Gioanni et al., 1999; Lambe et al., 2003; Disney et al., 2007; Zolles et al., 2009; Poorthuis et al., 2012; Aracri et al., 2013) as well as inhibitory (Xiang et al., 1998; Porter et al., 1999; Alkondon et al., 2000; Couey et al., 2007; Aracri et al., 2010) neurotransmission at both the pre- and post-synaptic level. These receptors also regulate dopaminergic neurons, which bears implications for tobacco addiction (Faure et al., 2014).

Interpreting the effects of nitrosamines in the brain requires considering their tonic actions in resting conditions as well as the phasic effects during transmitter release. The steady concentration of nicotine in the cerebrospinal fluid of smokers is close to the plasma levels (Berridge et al., 2010). Because the tonic extracellular concentrations of ACh in the brain is thought to be in the nanomolar range (Descarries et al., 1997), the steady effects of nitrosamines in the brain likely compete mostly with nicotine, whose levels depend on the smoking regimen. Considering that α4β2 nAChRs display a higher affinity for NNN, and that the latter tends to have a concentration higher than NNK, our results suggest that the inhibitory effect of NNN should prevail, in steady state conditions, irrespective of the nicotine level.

In contrast, in *bona fide* cholinergic synapses, on vesicle release, the ACh concentration in the synaptic cleft quickly reaches millimolar levels, thus activating the postsynaptic current within hundreds of μs. The subsequent current decay displays a kinetics that depends on tissue, largely because of the balance between intrinsic nAChR desensitization and ACh removal by acetylcholinesterase, but is generally complete within 5 to 20 ms (e.g., Magleby and Stevens, 1972; Chu et al., 2000). Our results (Figures 4B, 5B) suggest that the typical steady state concentrations of nitrosamines normally observed in blood are probably too low to affect classic postsynaptic currents activated by high ACh levels. However, it is possible that long exposures to nitrosamine compounds could affect the nAChR sensitivity and expression.

## Conclusions

The study of the interaction between nitrosamines and nAChRs is still in its infancy. Our results show that NNK behaves as a partial agonist of α4β2 nAChRs, whereas NNN is a receptor's inhibitor, with a relatively high affinity. In light of the above discussion, these properties may explain at least in part the effects produced by nitrosamines on neoplastic cell lines. However, to reach a full biological interpretation of the nitrosamines' roles *in vivo*, including those in the CNS, further studies are needed along the following lines. First, the effects of NNN and NNK should be tested on other nAChR subtypes, particularly α7. Second, whether nitrosamines induce long-term inactivation of nAChRs should be also analyzed. Finally, it will be necessary to ascertain the presence of different binding sites for these drugs on the receptor's protein.

## Funding

The present work was supported by the BML (Banca del Monte di Lombardia) Foundation to AB and the University of Milano-Bicocca (*Fondo di Ateneo per la Ricerca* to AB).

### Conflict of interest statement

The authors declare that the research was conducted in the absence of any commercial or financial relationships that could be construed as a potential conflict of interest.

## Abbreviations

CNS: central nervous system
DHβE: dihydro-β-erythroidine
nAChR: neuronal nicotinic acetylcholine receptor
EC_50high_ and EC_50low_: EC_50_ values for two-term Hill equations
HEK: human embryonic kidney
n_H_: Hill coefficient for one-term Hill equation
n_H1_, n_H2_: Hill coefficients for two-term Hill equation
NNK: 4-(methylnitrosamine)-1-(3-pyridyl)-1-butanone
NNN: N′-nitrosonornicotine
V_m_: membrane potential
V_rev_: reversal potential.
